# CGUFS: A clustering-guided unsupervised feature selection algorithm for gene expression data

**DOI:** 10.1016/j.jksuci.2023.101731

**Published:** 2023-10

**Authors:** Zhaozhao Xu, Fangyuan Yang, Hong Wang, Junding Sun, Hengde Zhu, Shuihua Wang, Yudong Zhang

**Affiliations:** a School of Computer Science and Technology, Henan Polytechnic University, Jiaozuo, Henan 454000, China; b Department of Gynecologic Oncology, The First Affiliated Hospital of Henan Polytechnic University, Jiaozuo, Henan 454000, China; c School of Computing and Mathematical Sciences, University of Leicester, Leicester LE1 7RH, UK

**Keywords:** Gene expression data, Clustering-guided, Unsupervised feature selection, *k*-means, Spectral clustering

## Abstract

**Aim:**

Gene expression data is typically high dimensional with a limited number of samples and contain many features that are unrelated to the disease of interest. Existing unsupervised feature selection algorithms primarily focus on the significance of features in maintaining the data structure while not taking into account the redundancy among features. Determining the appropriate number of significant features is another challenge.

**Method:**

In this paper, we propose a clustering-guided unsupervised feature selection (CGUFS) algorithm for gene expression data that addresses these problems. Our proposed algorithm introduces three improvements over existing algorithms. For the problem that existing clustering algorithms require artificially specifying the number of clusters, we propose an adaptive *k*-value strategy to assign appropriate pseudo-labels to each sample by iteratively updating a change function. For the problem that existing algorithms fail to consider the redundancy among features, we propose a feature grouping strategy to group highly redundant features. For the problem that the existing algorithms cannot filter the redundant features, we propose an adaptive filtering strategy to determine the feature combinations to be retained by calculating the potentially effective features and potentially redundant features of each feature group.

**Result:**

Experimental results show that the average accuracy (ACC) and matthews correlation coefficient (MCC) indexes of the C4.5 classifier on the optimal features selected by the CGUFS algorithm reach 74.37% and 63.84%, respectively, significantly superior to the existing algorithms.

**Conclusion:**

Similarly, the average ACC and MCC indexes of the Adaboost classifier on the optimal features selected by the CGUFS algorithm are significantly superior to the existing algorithms. In addition, statistical experiment results show significant differences between the CGUFS algorithm and the existing algorithms.

## 1 Introduction

The rapid development of biological sequencing technology has realized the automatic acquisition of large-scale gene expression data, which provides a new way for the pathogenesis and diagnosis of cancer and other diseases (Kang et al., 2023; Li et al., 2019; Pu et al., 2021; Xu et al., 2020). However, gene expression data are typically high-dimensional with a small size of samples, containing a large number of features unrelated to the disease of interest (Pu et al., 2021; Xu et al., 2022; Xu et al., 2021). Feature selection is a common method to solve the high-dimensional feature space and high feature redundancy of gene expression data. However, existing feature selection algorithms cannot handle gene expression data with missing class labels (Gao et al., 2021; Sun et al., 2023).

The challenges associated with obtaining labels for gene expression data have led to growing interest in unsupervised selection algorithms among researchers (Wang et al., 2020; Nie et al., 2018). These algorithms can be divided into three classes: filter, wrapper and embedded methods, depending on their level of independence from the clustering algorithms (Solorio-Fernández et al., 2020). Filter methods are independent of the clustering algorithms by ranking features and selecting the features with higher score (He et al., 2005; Ferreira and Figueiredo, 2012). However, this type of methods does not take into account the correlation between features, which means redundant features cannot be removed (Lee et al., 2018; Yan et al., 2020; Manbari et al., 2019). Conversely, wrapper methods reply on the clustering algorithm to evaluate the representative of features in the feature selection process (Dy and Brodley, 2004; Dutta et al., 2014), but it leads to over-reliance on the clustering algorithm, limiting the performance of feature selection (Cai et al., 2010; Shen et al., 2020).

Embedded methods usually integrate the feature selection process and the clustering algorithm, and automatically perform feature selection during the training process of the clustering algorithm (Mahendran and PM, 2022; Li et al., 2012). Many studies (Cai et al., 2010; Guo et al., 2017; Zhu et al., 2017; Guo and Zhu, 2018) have demonstrated that the local manifold structure of data contains crucial information, leading to the development of embedded methods based on the local manifold structure of data, such as multi-cluster unsupervised feature selection (MCFS) (Cai et al., 2010), triplet induced unsupervised feature selection algorithm (IUFS) (Guo et al., 2017), subspace clustering guided unsupervised feature selection algorithm (SCUFS) (Zhu et al., 2017) and dependence guided unsupervised feature selection algorithm (DGUFS) (Guo and Zhu, 2018). However, these algorithms only select features based on the correlation between features and pseudo-labels, which neglects the feature correlation and fails to eliminate redundant features (Yang et al., 2011).

Recently, there has been a trend towards integrating feature correlation analysis and redundancy analysis for feature selection (Du and Shen, 2015; Zhao and Liu, 2007b; Zhu et al., 2018; Yang et al., 2011). Unsupervised discriminative feature selection (UDFS) (Yang et al., 2011), unsupervised feature selection algorithm with adaptive structure learning(FSASL) (Du and Shen, 2015) spectral feature selection (SPFS) (Zhao and Liu, 2007b) and co-regularization unsupervised feature selection (CUFS) (Zhu et al., 2018) are examples of such algorithms. However, these algorithms have higher computational complexity and are not suitable for gene expression data (Lim and Kim, 2021; Li et al., 2019). To address this issue, some researchers (Zhao et al., 2022; Bandyopadhyay et al., 2014) have proposed using clustering algorithms to group features with high similarity into clusters, which effectively reduces the redundancy between features. However, clustering algorithms have their limitations, and their parameters need to be set in advance (Fränti and Sieranoja, 2019; Huang et al., 2019). Additionally, determining the appropriate number of features after grouping can also pose a challenge (Yan et al., 2020).

Building upon the discussion above, this paper proposes a clustering-guided unsupervised feature selection algorithm (CGUFS) for gene expression data. The proposed algorithm proceeds in three main steps. Firstly, all samples are grouped by the adaptive *k*-means algorithm, and each sample is assigned a corresponding pseudo-label. Next, all features are grouped by the spectral clustering algorithm, and each feature is assigned a corresponding group-label. Finally, potentially effective features and potentially redundant features in each feature group are determined based on the pseudo-labels of samples and the group-labels of features, and the number of features to be retained in each group is calculated using a feature group strategy. To demonstrate the effectiveness of the CGUFS algorithm, we conducted experiments on 7 high-dimensional datasets, using average result comparison, optimal value result comparison, and feature scale comparison. Additionally, we performed statistical comparison tests to determine whether significant differences exist between the CGUFS algorithm and the existing algorithms. Our contributions are summarized as follows:

(1) Our proposed CGUFS algorithm addresses the issue of requiring artificially specifying parameters in existing clustering algorithms by proposing an adaptive *k*-value strategy. Based on the working principle of elbow method, a change function is proposed, and the *k* value of *k*-means is determined adaptively by updating the change function iteratively.
(2) We propose a feature grouping strategy to address the problem of high redundancy among features in existing algorithms. Spectral clustering pairs are used to group all the features, which makes the intra-group feature correlation very high, but the inter-group feature correlation very low.
(3) For the problem that the existing algorithms cannot filter the redundant features, we propose two adaptive filtering strategies. Specifically, the potentially effective feature strategy is introduced to identify the features that need to be retained in each feature group, while the potentially redundant feature strategy is to identify redundant features that need to be removed in each group.
(4) For the problem that existing algorithms cannot self-adaptively determine which features need to be retained, we propose an adaptive retention strategy. The number of total features is used to calculate the number of features to be retained in the whole features, and the number of group features in the group is used to calculate the number of features to be retained in each group.

The remainder of this paper is organized as follows: Section 2 reviews the related works on unsupervised feature selection algorithms. Section 3 presents the details of the CGUFS algorithm. Experimental results, performance evaluation of the CGUFS algorithm, and comparison with other existing algorithms are presented in Section 4. Section 5 presents the experimental discussion. Finally, we draw conclusions and future work in Section 6.

## 2 Related works

Unsupervised feature selection (He et al., 2005; Dy and Brodley, 2004; Mahendran and PM, 2022) reduces the dimensionality of data by mapping data from a high-dimensional space to a low-dimensional space and removing redundant and irrelevant features. In this section, we provide an overview of classic unsupervised feature selection algorithms, which can be classed as filter (He et al., 2005), wrapper (Dy and Brodley, 2004), and embedded (Mahendran and PM, 2022) methods.

He et al. (2005) is a classical filter algorithm, which calculates the Laplacian score of each feature based on the principle that similar samples are closer. The lower the Laplacian score of the feature, the better the distinguishing ability of the feature. Similarly, Ferreira and Figueiredo (2012) proposed a filter algorithm based on association redundancy, which calculates the variance of features according to a correlation measure and ranks them in descending order. Then, the feature similarity measure is used to evaluate the features to quantify the redundancy between them, and top *k* features with the lowest redundancy are selected. Dy and Brodley (2004) used a forward search to search features and generate candidate features. Then, the expected maximum or the *k*-means algorithm is used to cluster the candidate features. Similarly, Dutta et al. (2014) employed a multi-objective genetic algorithm to search for features and then clustered the candidate features using the *k*-prototypes algorithm. Then the clustering effect of *k*-prototype is evaluated according to the minimum intra-cluster distance and maximum inter-cluster distance.

In recent years, researchers have embedded clustering algorithms into feature selection (Cai et al., 2010; Li et al., 2012). Cai et al. (2010) proposed the multi-cluster feature selection algorithm (MCFS), which uses spectral clustering to learn pseudo-labels of samples and solves the least squares problem with an L1 regularization term. Several top-scoring features are selected. This approach ensures that the selected features preserve the data cluster structure and cover the features of all possible clusters. Li et al. (2012) proposed the nonnegative discriminant feature selection algorithm (NDFS), which also employs spectral clustering to learn pseudo-labels of samples and completes feature selection in the learning of pseudo-labels. The combination of pseudo-labels and feature selection matrix enables the algorithm to select the most discriminative feature (Shang et al., 2021). To reduce redundancy and noise characteristics, *l*_2,1_-norm minimization constraint is added to the objective function to enforce the row sparsity of the feature selection matrix.

Qian and Zhai (2013) proposed the robust unsupervised feature selection algorithm (RUFS), which uses non-negative matrix decomposition to learn pseudo-labels of samples and performs feature selection by jointly minimizing *l*_2,1_-norm. This strategy can effectively deal with outliers and noise, and eliminates redundancy and noise features. Zhang et al. (2019) proposed the nonnegative Laplacian guided unsupervised feature selection algorithm, which combines the discriminant information of false labels with sub-space learning. The algorithm used non-negative Laplacian embeddings to generate pseudo-labels that improve classification ACC. Then, under the premise of preserving the local structure of data, the optimal features are selected through subspace learning guided by the discriminant information of pseudo-labels. To address the issue of noise and irrelevant features that reduce the quality of pseudo-labels generated by the clustering algorithm (Liu et al., 2018; Oliveira et al., 2022), Liu et al. (2016a) proposed the consensus guided unsupervised feature selection algorithm. The algorithm employs consensus clustering to learn pseudo-labels of samples. In addition, weighted *k*-means is proposed to provide theoretical support for the optimization of sample pseudo-labels.

Zhao and Liu (2007b) proposed a spectral analysis feature selection algorithm where the radial basis function kernel was used to establish the similarity matrix of data represented in the form of graph. Then the features are evaluated based on the spectrum of the constructed graph. As this algorithm is based on the kernel to build the similarity matrix, it can be used to select the continuous or binary features. Yang et al. (2011) proposed a unsupervised discrimination feature selection algorithm (UDFS), which uses batch processing to select discriminative features from the whole feature geometry. By classifying input data and using linear classifier for prediction, so the algorithm can utilize both discriminant information and feature relationships. The above works to transform the unsupervised problem into supervised feature selection based on sparse learning by clustering to generate pseudo-labels of samples (Yang et al., 2011; Zhao and Liu, 2007b; Tang et al., 2021; Wang et al., 2015). Different from the above works, Wang et al., 2015 embedded feature selection directly into the clustering algorithm through sparse learning. In addition, the multiplier alternate method is used to solve the optimization problem of the algorithm. Similarly, Zhu et al. (2018) proposed a collaborative regularization unsupervised feature selection algorithm. Joint *l*_2,1_-norm co-regularization is applied to multiple feature selection matrices to ensure that the selected features can maintain both data distribution and reconstruction.

The aforementioned pieces of work fail to fully consider the redundancy between features. In order to address this problem, Mitra et al. (2002) proposed a similarity-based unsupervised feature selection algorithm. Their approach involves calculating the similarity between features and combining features with low similarity. Since no search strategy is required, this approach is suitable for feature selection of large datasets. Similarly, He et al. (2017) proposed a decision graph-based unsupervised feature selection algorithm (DGFS). The algorithm calculated the local densities of features and selected those features with high local density as the representative features. Based on the redundancy and similarity between features measured by a defined discriminant distance, the decision graph score is used as the evaluation criterion for feature selection. Features with high decision graph scores constitute representative features. Experimental results show that their proposed algorithm is significantly better than the existing unsupervised feature selection algorithms.

In addition to the aforementioned methods, some researchers have proposed to select features by clustering features with high similarity into a group and calculating the redundancy within the group (Yan et al., 2020; Cheung and Jia, 2012). Cheung and Jia (2012) presented a feature clustering-based unsupervised feature selection algorithm that groups similar features into a cluster and selects the representative features of each group according to the redundancy within the group. This approach results in features that are representative of different groups, with low similarity between the selected features, and retaining the most data structure information with a fewest number of features. Similarly, Yan et al. (2020) proposed a feature clustering-based unsupervised feature selection algorithm, where the density clustering algorithm is first used to cluster the feature into multiple unrelated groups. Then, the most representative features from each group are selected to form a subset of candidate features.

Analysis of current clustering-guided unsupervised feature algorithms reveals two major challenges: 1) The reliability of feature selection results is directly impacted by the choice of *k* value in the sample grouping process. Moreover, feature clustering is a computationally complex NP-hard problem, which further complicates the selection process (Yan et al., 2020; Rezaee et al., 2021; Liu et al., 2016b). 2) How to select representative features from each feature group after clustering features is another significant challenge that must be addressed (Yan et al., 2020; Boutsidis et al., 2009).

## 3 Methodology

**Fig. 1:**
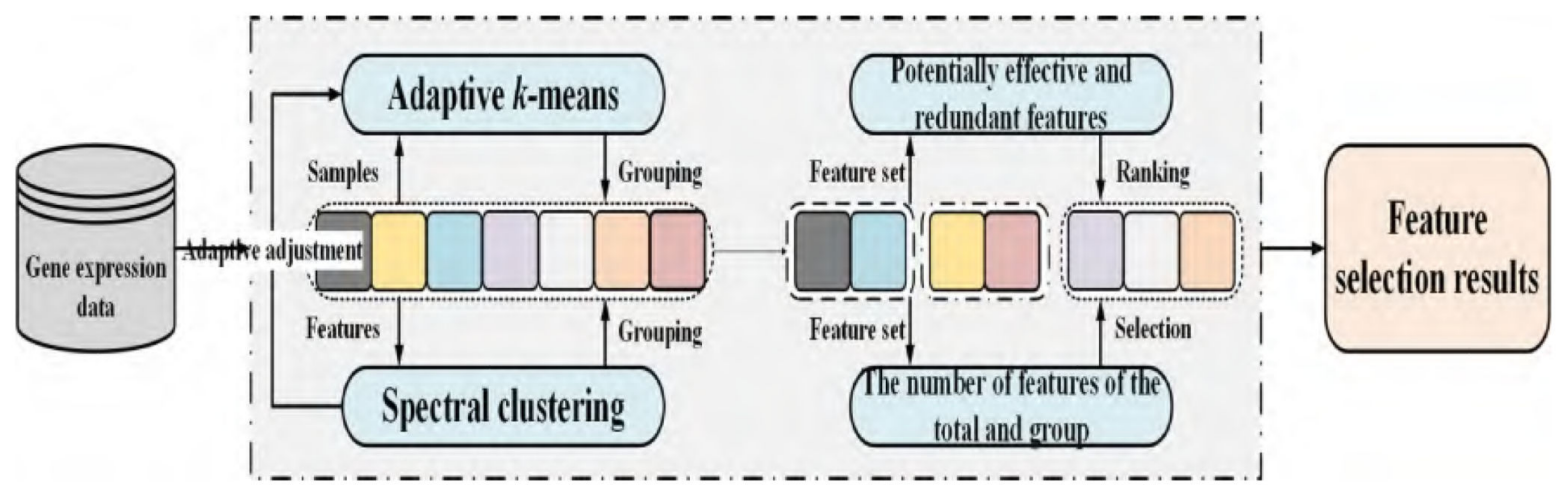
The flowchart of the CGUFS algorithm.

Fig. 1 shows the flowchart of the CGUFS algorithm. The CGUFS algorithm is divided into three parts: First, we introduce the proposed adaptive *k*-means algorithm that groups all samples and assigns a corresponding pseudo-label to each sample. Then, we use the spectral clustering algorithm to group all features and assign a corresponding group-label to each feature. Final, we use the potentially effective feature and potentially redundant feature strategies for feature selection.

### 3.1 Symbol definition

Given a dataset *X* = {*x_i_*, *f_j_*}, *i* = 1, 2, … , *n*; *j* = 1, 2, … , *d*, *n* and *d* represent the number of samples and the number of features in the dataset, respectively. Where, *x_i_* represents the *i* th sample of the dataset and *f_j_* represents the *j* th feature of the dataset. For ease of understanding, Table 1 presents the symbols and definitions used in this paper.

### 3.2 An adaptive k-means sample grouping algorithm

Supervised feature selection algorithms cannot be directly applied when label information of samples is lacking. To address this issue, we propose using a clustering algorithm to group samples and assign a pseudo-label to each sample. Then, supervised feature selection can be carried out guided by the learned pseudo-labels.

*k*-means is a partition-based clustering algorithm (Bishnu and Bhattacherjee, 2011), which uses a similarity criterion to divide samples into several clusters based on spatial distance. Firstly, *k* samples from the dataset *X* are randomly selected as the initial cluster centers. The distance *d*(*x_i_*, *Center*_*l*1_) between a sample *x_i_* and a cluster center *Center*_*l*1_ is calculated according to Eq. (1):

(1) $$\begin{array}{l} d\left(x_{i},Center_{l1}\right)=\sqrt{\sum_{j=1}^{d}\left(x_{ij}-Center_{l1j}\right),}j=1,2,\ldots ,d;l1\\ \;=1,2,\ldots ,k \end{array}$$

where, *d*(*x_i_*, *Center*_*l*1_) is the Euclidean distance between *x_i_* and *x_i_* = (*x*_*i*1_, *x*_*i*2_, …, *x_id_*) is a sample in the *N*-dimensional feature space, and *Center*_*l*1_ = (*Center*_*l*11_, *Center*_*l*12_, … , *Center*_*l*1*d*_) is a cluster center in the *N*-dimensional feature space.

Each sample is assigned to the nearest cluster based on its distances to all cluster centers. Subsequently, a new cluster center is formed according to the average value of sample points in each group, and this process is repeated until the criterion function of Eq. (2) converges.

(2) $$J=\sum_{l1=1}^{k}\sum_{x_{i}\in Center_{l1}}{\left|x_{i}-m_{l1}\right|}^{2},i=1,2,\cdots ,n;l1=1,2,\cdots ,k$$

where, *J* is the sum of squared errors of all samples, *x_i_* is a sample point in the *d*-dimensional feature space, and *m*_*l*1_ is the cluster center of group *Center*_*l*1_. Groups generated according to this criterion tend to be independent and compact.

The *k*-means algorithm requires specifying the number of clusters (*k* value) in advance, which limits the rationality of *k*-means to a certain extent (Liu et al., 2016b; Bishnu and Bhattacherjee, 2011). Elbow method is a common method to determine the value of *k*, its core idea can be described as, With the increase of the number of groups *k*, the sample division will be more detailed, the degree of aggregation of each group will gradually increase, then the sum of squared errors (SSE) will naturally become smaller. The relationship between SSE and *k* is the shape of an elbow, and the *k* value corresponding to the elbow is the real number of groups of data.

The elbow method has high computational efficiency and is easy to understand compared with other *k* selection algorithms. However, the elbow method mainly has the following problems (Liu and Deng, 2020; Racolte et al., 2022): (1) When the *k* value is lower than the real group, the SSE may decrease slowly, that is, the ”elbow point” is not obvious. (2) When the k value is higher than the real group, the SSE may rise, that is, the ”elbow point” is negative. (3) The existing elbow method needs to manually select the ”elbow point”, and cannot automatically select the ”elbow point”.

When the *k* value is less than the number of real groups, the SSE decreases significantly with the increase of the *k* value. However, when *k* is greater than the number of real groups, the SSE will slowly decrease with the increase of *k* value. According to this characteristic, we propose a variation function to find the optimal *k* value by calculating the difference of the SSE after grouping according to Eq. (3).

(3) $$\Delta J=\left|J_{l}-J_{l+1}\right|,l=1,2,\cdots ,k_{1}$$

When the elbow point is not obvious, determining the *k* value can be challenging. According to the principle of elbow method, the point where the SSE decreases the most is selected as the optimal *k* value. In particular, this corresponds to the maximization of the sum of squared errors as defined by Eq. (4).

(4) $$Max\left\{\Delta J\right\},l=1,2,\cdots ,k_{1-1}$$

When the elbow is negative, the k value is already higher than the real group. Similarly, according to the characteristics of the elbow method, we select the point before the negative elbow as the optimal k value, as shown in Eq. (5).

(5) $$IF\left\{\Delta J\right\},l=1,2,\cdots ,k_{1-1}$$

Algorithm 1 presents an adaptive *k*-means sample grouping algorithm. The pseudo-label of each sample is determined by adaptive determination of *k* value, and the correlation between features and features and between features and pseudo-labels is calculated, so as to facilitate the subsequent retention or filtering of such features. Specifically: Steps 1–7 are the initial sample grouping process, in which we group all samples using the *k*-means algorithm and assign each sample a corresponding pseudo-label. Steps 8–11 are the final sample grouping process, in this stage, we use the change function to adaptively determine the *k* value and assign the final pseudo-label to each sample.

Algorithm 1 **An adaptive *k*-means sample grouping algorithm**

**Require:** Sample subsets: *X* = (*x*_1_, *x*_2_, …, *x_n_*), Maximum number of iterations: *k*_1_

**Ensure:** Sample grouping: $X=\left(X_{1},X_{2},\cdots ,X_{u_{1}}\right)$

      **for** *i* = 1, 2, …, *n* **do**

  1: Randomly select *l*1 samples from the sample subsets *X* as the initial cluster centers: *Center*_*l*1_.

  2: **repeat**

      3: According to Eq.(1), calculate the *d*(*x_i_*, *Center*_*l*1_) between *x_i_* and *Center*_*l*1_.

      4: According to *d*(*x_i_*, *Center*_*l*1_), *x_i_* is assigned to the nearest cluster center *Center*_*l*1_

      5: According to Eq.(2), update all clustering centers *Center*_*l*1_.

  6: **until** All cluster centers have no changes

  7: Save the *u*_1_-th sample group *X* and the sum of squared error $J_{u_{l}}$

   **for** *u*_1_ = 1, 2, …, *k*_1_ **do**

   8: According to Eq.(3), calculate the sum of squared error of two groups errors and save them in $\Delta J_{u_{1}}$

**if then**

   9: According to Eq.(5), select the *u*_1_-1 th cluster as the optimal *k* value.

10: **return** Sample grouping: $X=\left(X_{1},X_{2},\cdots ,X_{u_{1}-1}\right)$

**else**

11: According to Eq.(4), select the *i*-th cluster as the optimal *k* value

12: **return** Sample grouping: $X=\left(X_{1},X_{2},\cdots ,X_{u_{1}}\right)$

### 3.3 A spectral clustering feature grouping algorithm

To address the issue of highly correlated feature groups and redundant features in high-dimensional data, it is necessary to group features in order to identify mutually redundant feature groups. However, traditional *k*-means algorithms are typically designed to find spherical structures and cannot capture non-convex structures in high-dimensional data.

Spectral clustering is based on graph theory, which transforms the sample clustering into the partitions of a weighted undirected graph where vertices represent samples and weighted edges represent similarity beteween samples. Different from general spectral clustering, we propose a spectral clustering feature grouping algorithm.

Firstly, the feature clustering problem is transformed into a feature graph partition problem by taking features as vertices and similarity between features as edge weight. The similarity matrix *W* = *R*^*m*×*m*^ is established according to the similarity between any two points. The similarity between any two points is defined as follows:

(6) $$w_{ij}=\exp\left(-\frac{{\left\|f_{i}-f_{j}\right\|}^{2}}{\sigma^{2}}\right)$$

where, *σ* is a scaling parameter, *f_i_* and *f_j_* are the *i*-th and *j*-th features, respectively, and ||*f_i_* − *f_j_*||^2^ is the Euclidean distance between the two features.

The goal of the Ncut graph is to cut a graph *G*(*V*, *E*) into *k* unconnected sub-graphs. The set of *k* sub-graph points is *A*_1_, *A*_2_, … , *A_k_*, which satisfies *A_i_* ∩ *A_j_* = ∅ and *A*_1_ ∪ *A*_2_ ∪ … ∪ *A_k_* = *V*. For the set of any two sub-graph points *A*, *B* ⊂ *V*, *A* ∩ *B* = ∅, the tangent graph weight between *A* and *B* is:

(7) $$W\left(A,B\right)=\sum_{i\in A,j\in B}w_{ij}$$

The Ncut graph is constructed based on weights. The goal is to reduce the cost of cutting the graph and maximize the sum of weights in the cluster. Then, the workflow of the Ncut graph can be described as follows:

Let’s first introduce the indicator vector *h_j_*. where *h_j_* ∈ {*h*_1_, *h*_2_, …, *h_k_*}, *j* = 1, 2, …, *k*, *h_j_* is a column vector of *N*-dimensional feature space,

(8) $$h_{ij}=\{\begin{array}{l} \frac{1}{\sqrt{vol\left(A_{j}\right)}}v_{i}\in A_{j}\\ 0v_{i}\in A_{j} \end{array}$$

where, *A*_1_, *A*_2_, … , *A_k_* are *k*-class divisions of the Ncut graph. The spectral clustering algorithm based on multipath cutting can meet the minimum Ncut standard by analyzing piecewise constant feature vectors. Eq. (8) is the piece wise constant feature vector extracted from feature vector. Suppose *H* is a matrix composed of *K* column vectors, then:

(9) $$h_{i}^{T}Lh_{i}=cut\left(A_{i},\bar{A_{i}}\right)/vol\left(A_{i}\right)$$

Eq. (9) is the piecewise constant feature vector extracted from the feature vector. Suppose *H* is a matrix composed of a *k* column vectors, minimizing Ncut ultimately translates to minimizing:

(10) arg min︸Htr(HTD1/2LD1/2H)s.t..HTH=I

The *k*-th smallest eigenvalue and its corresponding eigenvector of the normalized Laplacian matrix are obtained. The standardized processing formula is defined as follows:

(11) $$L=D^{-1/2}WD^{-1/2}$$

where, $D_{ij}=\sum_{j=1}^{n}W_{ij}$ is the degree matrix. The matrix *V* = (*v*_1_, *v*_2_, …, *v_k_*) ∈ *R*^*n*×*k*^ is the eigenvector corresponding to the first *k* maximum eigenvalues of Laplace matrix *L*. Normalized the row vector of *V*, the unit length vector matrix *Y* ∈ *R*^*n*×*k*^ is obtained, where:

(12) $$Y_{ij}=Y_{ij}/{\left(\sum_{j}V_{ij}^{2}\right)}^{1/2}$$

Each row of data of *Y* is regarded as a data point in the *R^k^* space, and each feature point in *Y* is divided into *k*_2_ class by *k*-means algorithm. If the *i*-th row of matrix *Y* classified into the *j*(*j* ∈ [1, *k*]) class, the feature *F_i_* is divided into the *j*-th class.

Algorithm 2 presents a spectral clustering feature grouping algorithm. By grouping features, highly related features are grouped together to facilitate subsequent retention and filtering. Specifically: Steps 1–6 are the eigenmatrix construction process. By constructing a standardized Laplacian matrix, the smallest *k* eigenvalues are selected to form the corresponding eigenmatrix. Step 7–8 are the feature grouping process. In this stage, we call Algorithm 1 to group the feature matrix until the maximum number of iterations *k*_2_ is reached, and each feature is assigned a corresponding group label.

Algorithm 2 **A spectral clustering feature grouping algorithm**

**Require:** Features: *F* = {*f*_1_, *f*_2_, … , *f_d_*}, Maximum number of iterations:

   *k*_2_

**Ensure:** Feature grouping: $F=\left\{F_{1},F_{2},\cdots ,F_{u_{2}}\right\}$

  1: According to Eq.(6), the similarity matrix *W* between features is constructed.

  2: According to *W*, construct degree matrix *D* and Laplace matrix *L*.

  3: **repeat**

  4:   The eigenvector corresponding to the first *k* eigenvalues is found.

  5:   The matrix is constructed according to eigenvector, and the row vectors of matrix are normalized.

  6: **until** All of the row vectors are normalized

      **for** *u*_2_ = 1, 2, … , *k*_2_ **do**

  7: Let *y_i_* ∈ *R^k^*, *i* = 1, 2, … , *n* is the *i*-th row of *Y*.

  8: Call Algorithm 1 to group *y_i_* and output the optimal *k* value.

       **return** Feature grouping: $F=\left\{F_{1},F_{2},\cdots ,F_{u_{2}}\right\}$.

### 3.4 A clustering-guided unsupervised feature selection algorithm

After feature grouping, the intra-group feature correlation is high, while the inter-group feature correlation is low. To identify redundant features, correlation between features is often considered as a criterion, where two features are deemed redundant if they are highly correlated. In this section, we formally define redundant features based on the Markov blanket, which satisfies the following conditions:

**Definition 1** (Markov blanket): Given the feature set *F_j_*, *j* = 1, 2, … , *k* after feature grouping and the pseudo-label clusters after sample grouping, select the feature $F_{j}^{p}\left(F_{j}^{p}\notin M_{l}\right)$ in *F_j_*, if $F_{j}^{p}$ satisfies the constraint conditions of Eq. (13). Then *M_l_* is the Markov blanket of $F_{j}^{p}$.

(13) $$\left(F_{j}^{p}⊥\left\{F_{j}-M_{l}-\left\{F_{j}^{p}\right\},Cluster\right\}|M_{l}\right)M_{l}\in F_{j}$$

where, the symbol “⊥” represents independent, “|*M_l_*” means under the condition of given *M_l_*. According to Definition 1, for *F_j_* and Cluster, if *M_l_* ∈ *F_j_* is the Markov blanket of $F_{j}^{p}$, then $F_{j}^{p}$ is a redundant feature in *F_j_*. However, this completely conditional independent relation is NP-hard problem. Therefore, Markov blanket conditions need to be approximated as following:

**Definition 2** (Approximate Markov blanket): Given the features $F_{j}^{p}$ and $F_{j}^{p}$ in the feature set *F_j_*, the information gains between $F_{j}^{p}$, $f_{j}^{q}$ and the pseudo-label cluster are *IG*($F_{j}^{p}$, *Cluster*) and *IG*($F_{j}^{q}$, *Cluster*), and the mutual information between $F_{p}^{j}$ and $F_{j}^{q}$ is *IG*($F_{j}^{p}$, $F_{j}^{q}$). If $F_{j}^{p}$ and $F_{j}^{q}$ satisfy the constraints of Eq. (14), then $F_{j}^{p}$ is the approximate markov blanket of $F_{j}^{q}$.

(14) $$\{\begin{array}{l} IG\left(F_{j}^{p},Cluster\right)>IG\left(F_{j}^{q},Cluster\right)\\ IG\left(F_{j}^{q},Cluster\right)<IG\left(F_{j}^{p},F_{j}^{q}\right) \end{array}$$

$F_{j}^{p}$ in *F_j_* is selected as the main feature, and all redundant features from *F^p^_j_* as the main feature is removed according to Eq. (14). However, blindly removing redundant features may lead to the loss of important informative features. In addition, the correlation between features and pseudo-labels may also change after feature grouping.

Therefore, we propose two feature selection strategies, namely potential effective features and potential redundant features. Firstly, to calculate the correlation of features with pseudo-labels, and we give the relevant definition of symmetric uncertainty.

**Definition 3** (Symmetric uncertainty): For feature *f_j_* and *Cluster*, symmetric uncertainty can be used to measure the degree of dependence between them, and can represent the information content they share. It can also be understood as the degree to which the uncertainty of one feature is reduced when the other feature is known. Then, the symmetric uncertainty *SU*(*f_j_*, *Cluster*) of features can be described as:

(15) $$SU\left(f_{j},Cluster\right)=\frac{2IG\left(f_{j},Cluster\right)}{H\left(f_{j}\right)+H(Cluster)}$$

where, *IG*(*f_j_*, *Cluster*) represents the information gain between *f_j_* and *Cluster*. The greater the information gain, the stronger the correlation between the representation feature and the *Cluster*, and the stronger the distinguishing ability. *H*(*f_j_*) and *H*(*Cluster*) represent the information entropy of *f_j_* and *Cluster*, respectively.

**Definition 4** (Potential effective features): Given the features $F_{j}^{q}$ and $F_{j}^{p}$ in the feature grouping *F_j_*, with $F_{j}^{p}$ selected as the main feature, the information gain of $F_{j}^{q}$, $F_{j}^{p}$ and *Cluster* is *IG*($F_{j}^{q}$, *Cluster*) and *IG*($F_{j}^{p}$, *Cluster*) respectively. If the constraints of Eq. (16) are satisfied, then $F_{j}^{q}$ is the potential effective features to be retained in *F_j_*, and the set of Potential effective features $F_{j}^{q}=\left\{F_{j}^{q1},F_{j}^{q2},\cdots ,F_{j}^{qn}\right\}$ is the output.

(16) $$\{\begin{array}{l} IG\left(F_{j}^{p},Cluster\right)>IG\left(F_{j}^{q},Cluster\right)\\ IG\left(F_{j}^{q},Cluster\right)>IG\left(F_{j}^{p},F_{j}^{q}\right) \end{array}$$

**Definition 5** (Potential redundancy features): Given the features $F_{j}^{r}$ and $F_{j}^{p}$ in the feature grouping *F_j_* with $F_{j}^{p}$ selected as the main feature, the information gain between $F_{r}^{j}$, $F_{p}^{j}$ and *Cluster* are *IG*($F_{j}^{r}$, *Cluster*) and *IG*($f_{j}^{p}$, *Cluster*), respectively. If the constraints of Eq. (16) are satisfied, then $f_{j}^{r}$ is the potential redundant features to be removed in *F_j_*, and the output is the Potential redundant features $F_{j}^{r}=\left\{F_{j}^{r1},F_{j}^{r2},\cdots ,F_{j}^{rn}\right\}$.

From Definitions 4 and 5, the set of potential effective features and potential redundant features in each feature group can be obtained. Then according to Definition 3, the symmetric uncertainty values of all the features in each feature group are calculated and ranked in descending order. However, how many features should be retained from different feature groups is still a problem to be solved. Therefore, we propose a feature-based grouping weight strategy:

**Definition 6** (Number of total features): Assign the corresponding pseudo-label *Cluster*(*x_i_*, *Center*_*l*1_) to each sample *x_i_* according to Algorithm 1, and then calculate the symmetric uncertainty value of *f_j_* as *SU*(*f_j_*, *Cluster*) according to Definition 2. Then, the number of total features to be retained before feature grouping is $N_{new}=\beta \times \sum_{j=1}^{d}\left|f_{j}\right|$. Where, is used to calculate the number of features in *F*, and *β* is the percentage of the number of features retained in order.

**Definition 7** (Number of group features): Assign each feature *f_j_* the corresponding group-label *Cluster*(*f_j_*, *Center*_*l*2_) according to Algorithm 2, and then assign the *SU*(*f_j_*, *Cluster_j_*) of *f_j_* to the corresponding group *SU*($F_{j}^{p}$, *Cluster_j_*). Then, the number of group features to be retained after feature grouping is $N_{j\_new}=\sum_{p=1}^{k_{p}}SU\left(F_{j}^{p}\right)/\sum_{j=1}^{d}SU\left(f_{j}\right)\times N_{new}$. Where, $F_{j}^{p}$ refers to the feature that *f_j_* is assigned to the *j*-th group after feature grouping.

Algorithm 3 presents a clustering-guided unsupervised feature selection algorithm. Specifically: Steps 1–3 are the sample grouping process. In this stage, we call Algorithm 1 to obtain the pseudo-label of the sample, which provides support for the subsequent calculation of the correlation between global feature and feature, and between feature and pseudo-label. Steps 6–8 are the feature grouping process. In this stage, we call Algorithm 2 to obtain the group label of the feature, which provides support for the subsequent calculation of the correlation degree between the feature and the feature, and between the feature and the pseudo-label. Steps 9–11 are the feature selection process. In this stage, we use potentially effective features and potentially redundant feature strategies to calculate the features to be retained and deleted for each group, and adaptively determine the number of features to be retained according to the total number of features.

Algorithm 3 **A clustering-guided unsupervised feature selection algorithm**

**Require:** Dataset: *X* = {*x*_1*M*_, *x*_2*M*_, … , *x_nd_*}, Maximum number of iterations: *k*_1_, *k*_2_

**Ensure:** Dataset after feature selection:

$$X\prime =\left\{x_{1d_{new}},x_{2d_{new}},\cdots ,x_{nd_{new}}\right\}$$

 1: **repeat**

 2:  Call Algorithm 1 to group samples *x_i_* and assign corresponding pseudo-labels: *Cluster*(*x_i_*, *Center*_*l*1_).

 3: **until** Reaches the maximum number of iterations *k*_1_

 4: According to Definition 6, calculate the number of features to be retained before feature grouping.

 5: According to the *Cluster*(*f_j_*, *Center*_*l*1_), calculate the symmetric uncertainty value of the feature *f_j_*:

 6: repeat

 7:  Call Algorithm 2 to group feature *f_j_* and assign corresponding group-label: *Cluster*(*f_j_*, *Center*_*l*2_).

 8: **until** Reaches the maximum number of iterations *k*_2_

 9: According to Definition 4, calculate the potential effective features in feature group after feature grouping, and output.

 10: According to Definition 5, calculate the potential redundant features in feature group after feature grouping, and output.

 11: According to Definition 7, the number of features to be retained in each feature group is calculated.

   **return** Dataset after feature selection:

$$X\prime =\left\{x_{1d_{new}},x_{2d_{new}},\cdots ,x_{nd_{new}}\right\}$$

### 3.5 Algorithm complexity analysis

The time complexity and space complexity of CGUFS algorithm are divided into sample grouping, feature grouping and feature selection. In the sample grouping stage, the time complexity of adaptive *k*-means is *O*(*T*_1_*k*_1_) and the space complexity is *O*(*S*_1_*k*_1_). Where, *k*_1_ is the number of iterations of the final grouping. *T*_1_ and *S*_1_ are the time and space consumed by the initial grouping respectively. In the feature grouping stage, the time complexity of spectral clustering is *O*(*T*_1_*T*_2_*k*_2_) and the space complexity is *O*(*S*_1_*S*_2_*k*_2_)). Where, *k*_2_ is the number of iterations in the final group. *T*_1_ and *S*_1_ are the time and space consumed to construct the matrix, respectively.

In the feature selection stage, the time and space consumed by calling Algorithm 1 and Algorithm 2 are mainly used for sample grouping and feature grouping, and other time and space are negligible. In summary, the time complexity of CGUFS is *O*(*T*_1_(*k*_1_ + *T*_2_*k*_2_)). The space complexity is *O*(*S*_1_(*k*_1_ + *S*_2_*k*_2_)). The time complexity and space complexity of the CGUFS algorithm are both raised to the third power, while the time complexity and space complexity of the classical algorithm (Such as IUFS (Guo et al., 2017), etc) are at least raised to the fourth power, or even higher.

## 4 Experimental evaluations

### 4.1 Experiment datasets

To verify the effectiveness of the CGUFS algorithm, we conduct experiments on 7 high-dimensional datasets from diverse domains. Specifically, ALLAML, GLIOMA and Colon are gene expression datasets. WrapAR10P, wrapPIE10P and Yale are image datasets. Madelon is a synthetic dataset. The details of 7 high-dimensional datasets are shown in Table 2, where Feature, Sample, and Class denote the number of features, the number of samples and the number of classes, respectively.

### 4.2 Experiment settings

To evaluate the performance of the CGUFS algorithm, we compared it against several unsupervised feature selection algorithms, including Laplacian unsupervised feature selection algorithm (LS) (He et al., 2005), discrimination unsupervised feature selection algorithm (UDFS) (Yang et al., 2011), multi-cluster unsupervised feature selection (MCFS) (Cai et al., 2010), and adaptive structure learning unsupervised feature selection algorithm (FSASL) (Zhao and Liu, 2007a), triplet induced unsupervised feature selection algorithm (IUFS) (Guo et al., 2017), subspace clustering guided unsupervised feature selection algorithm (SCUFS) (Zhu et al., 2017) and dependence guided unsupervised feature selection algorithm (DGUFS) (Guo and Zhu, 2018). For all the algorithms, we fix the value of *k*, which specifies the size of the neighborhood, to 5 for all the datasets. The bandwidth parameter of the Gaussian kernel in LS needed to be tuned, while the regularization parameter required tuning for MCFS and UDFS.

In the experiment, classifiers including C4.5 and Adaboost are used to conduct 10-fold crossover test on the dataset after feature selection, and the results of accuracy (ACC), Recall, F-measure and matthews correlation coefficient (MCC) indexes are recorded. Among them, MCC is an evaluation metric for imbalanced data, which can avoid the defect that ACC is not suitable for high-dimensional data and F-measure mainly emphasizes classifier’s recognition ability of positive samples. The C4.5 classifier is first used to conduct the test on the different features selected by all the algorithms. Then, the C4.5 classifier and the Adaboost classifier are used to conduct the test on the optimal features selected by all algorithms. Finally, we report the feature scale (FS) of the C4.5 and Adaboost classifiers on the optimal set of selected features for comparison. In addition, Wilcoxon and Friedman tests are utilized to verify whether there are significant differences between the CGUFS algorithm and the existing algorithms.

### 4.3 Average experimental results

**Fig. 2:**
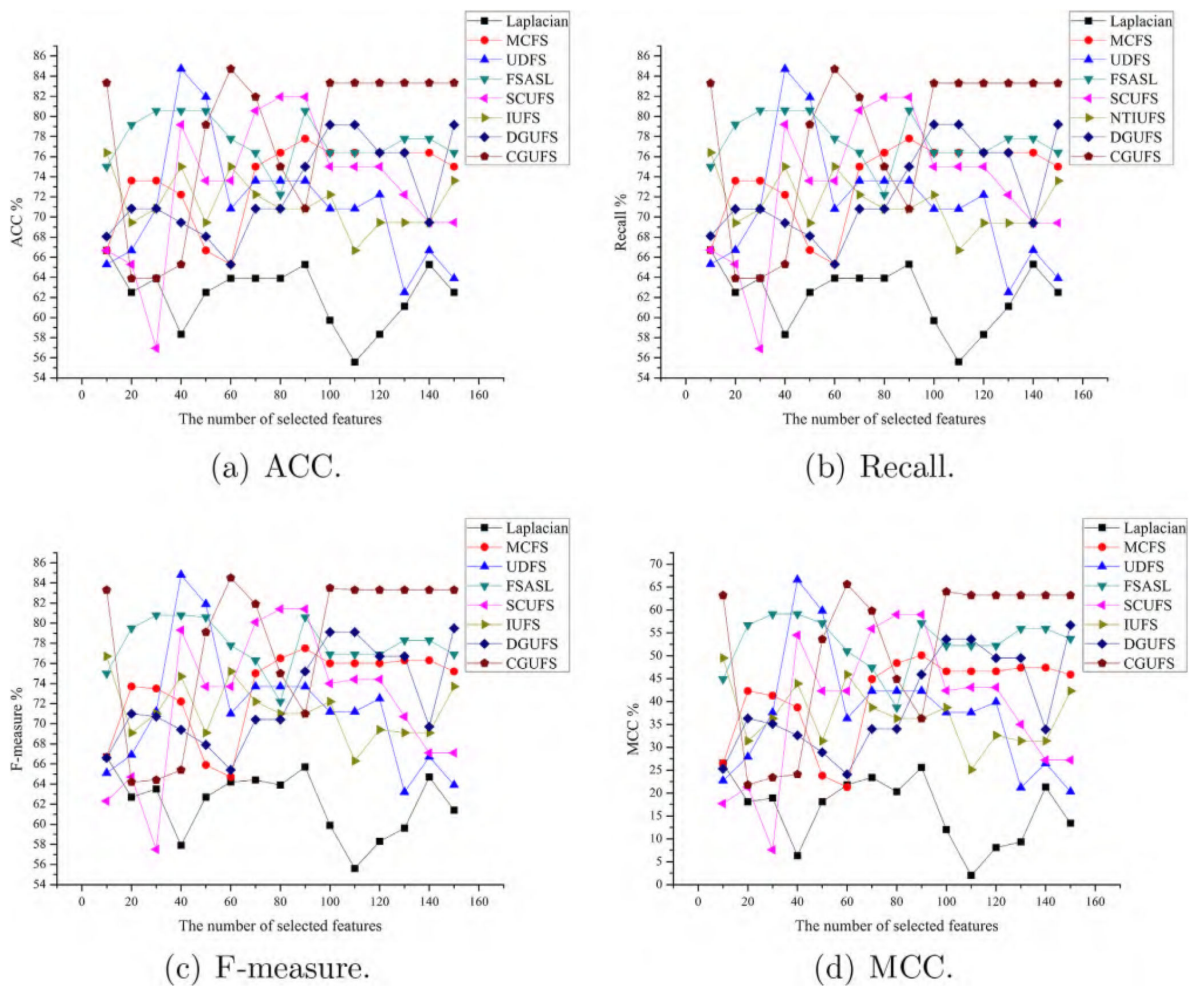
The performance of the C4.5 classifier on different sets of features selected by different algorithms on the ALLAML dataset.

**Fig. 3:**
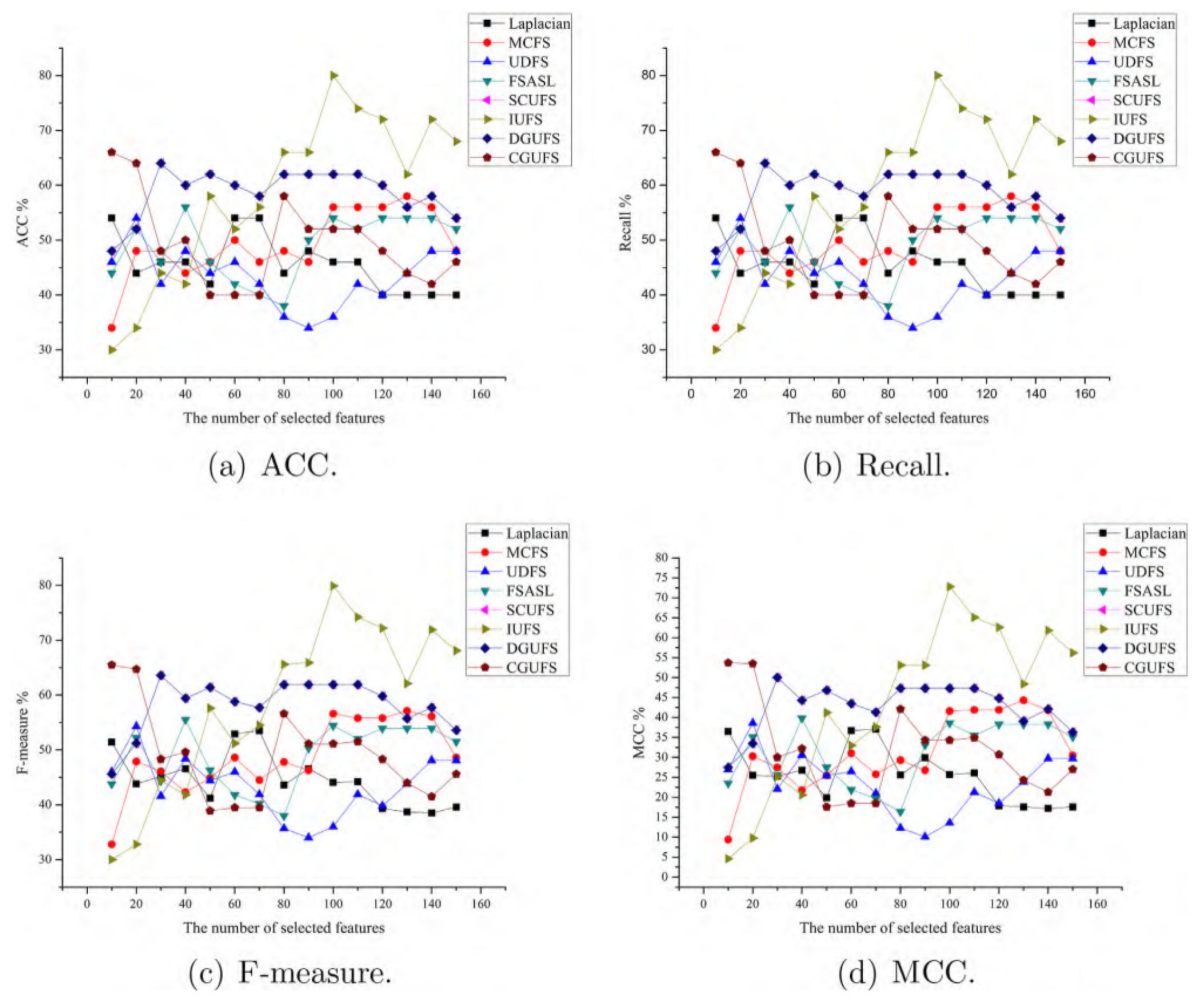
The performance of the C4.5 classifier on different sets of features selected by different algorithms on the GLOMA dataset.

**Fig. 4:**
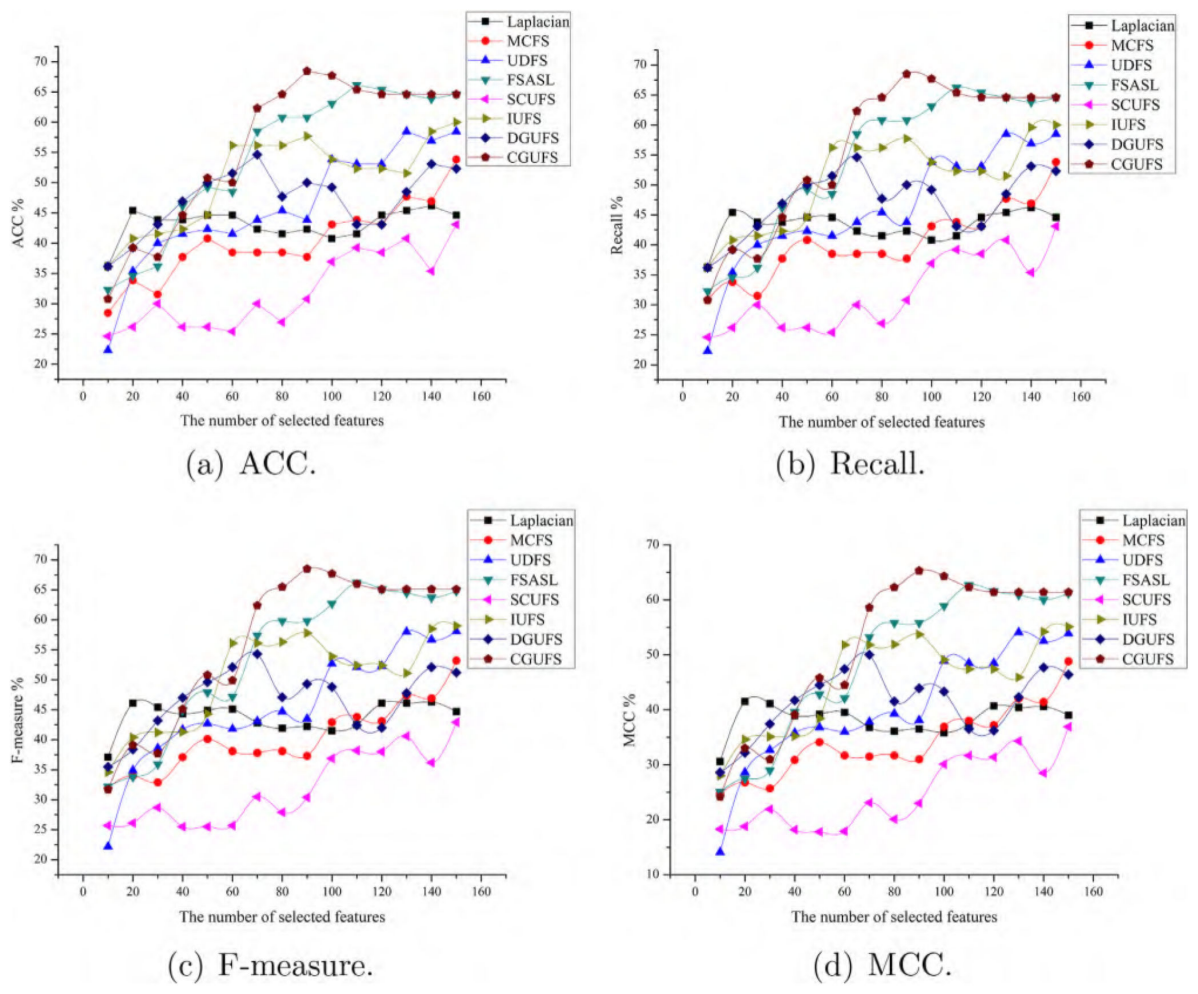
The performance of the C4.5 classifier on different sets of features selected by different algorithms on the warpAP10P dataset.

**Fig. 5:**
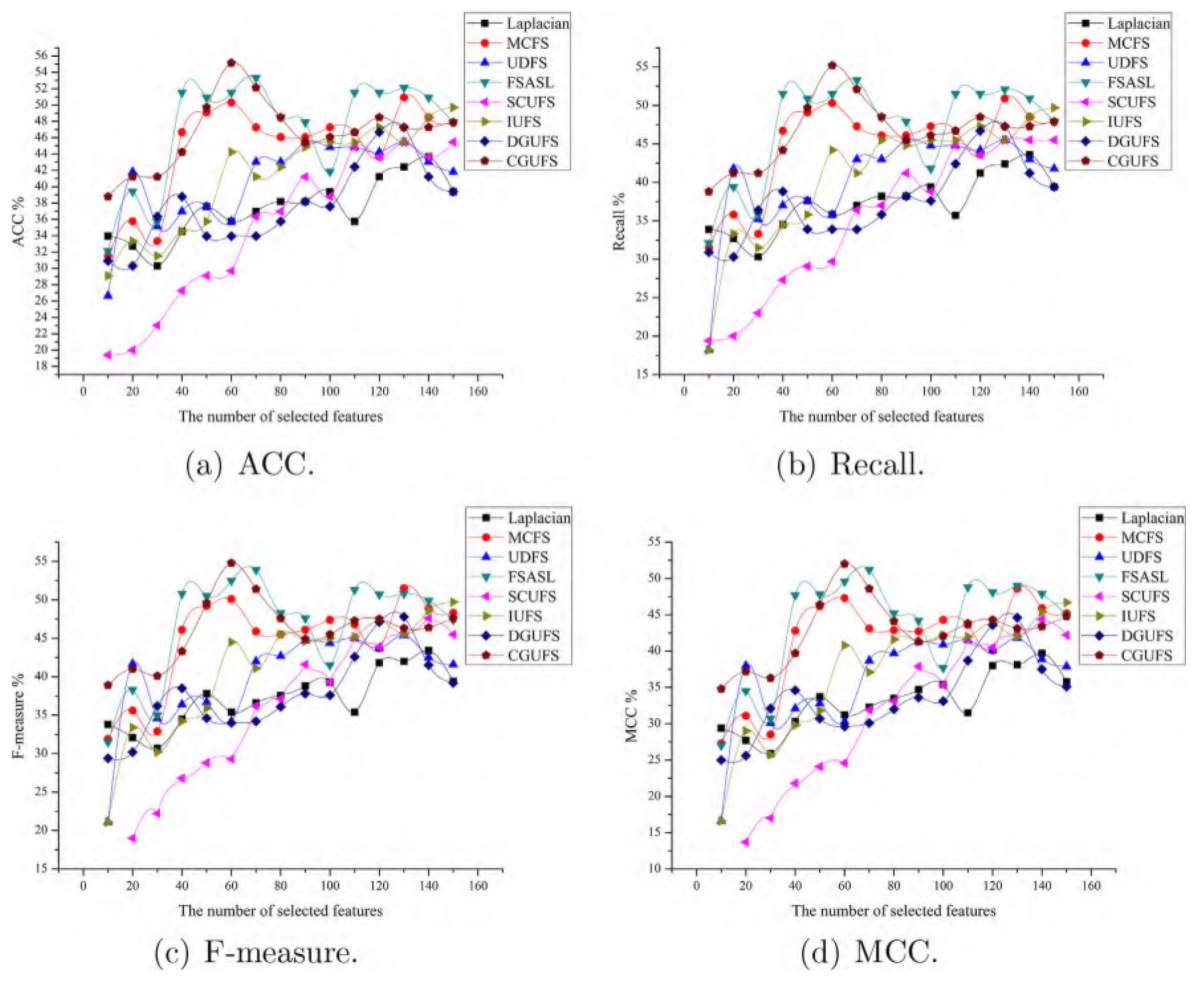
The performance of the C4.5 classifier on different sets of features selected by different algorithms on the Yale dataset.

To evaluate the performance of the CGUFS algorithm, we conduct experiments on four datasets, namely ALLAML, GLIOMA, warpAP10P, and Yale. The C4.5 classifier is used to test the features selected by all feature selection algorithms, and the evaluation metrics, including ACC, Recall, F-measure, and MCC, were reported. The results of the C4.5 classifier on the aforementioned datasets are shown in Fig. 2–5, respectively.

The results presented in Fig. 2 demonstrate the superiority of the CGUFS algorithm in terms of classification performance compared to Laplacian, FSASL, and SCUFS algorithms. The classification performance on the features selected by FSASL, SCUFS, and UDFS algorithms is moderate, while that of Laplacian, DGUFS, and MCFS algorithms is the poorest. It is worth noting that when the number of selected features is small, the performance of C4.5 classifier on the features selected by the UDFS algorithm slightly outperforms that of the CGUFS algorithm.

Fig. 3 shows that the classification performance on the features selected by the CGUFS algorithm is worse than that of the IUFS algorithm. However, when the number of selected features is relatively small, the classification performance on the features selected by IUFS algorithm is poor. In this case, the classification performance on the features selected by the CGUFS algorithm is better than the IUFS algorithm. Overall, the CGUFS algorithm achieves better classification performance with a smaller number of features.

Fig. 4 shows that features selected by the CGUFS algorithm lead to the best classification performance, followed by the features selected by FSASL, MCFS and IUFS algorithms, while the features selected by UDFS, DGUFS and Laplacian algorithms lead to the worst classification performance. Similarly, when a relatively large number of features are selected, the classification performance on the features selected by FSASL algorithm is better than that of the CGUFS algorithm.

According to Fig. 5, the performance of the C4.5 classifier on the features selected by the CGUFS algorithm are significantly better than the existing algorithms. When the number of selected features is less than 60, the features selected by the CGUFS algorithm lead to a better classification performance. When the number of selected features are greater than 60, the features selected by FSASL and IUFS algorithms lead to better classification performance. Overall, the classification performance is better with a smaller number of features selected by the CGUFS algorithm.

Comprehensive analysis of Fig. 2–5 demonstrates that the quality of selected features by the CGUFS algorithm is better than the existing algorithm on most datasets. IUFS algorithm has the best feature selection effect among existing algorithms. However, Laplacian algorithm has the worst feature selection effect among existing algorithms. In addition, for different datasets, there are obvious differences in the classification effect of different feature subsets. For example, in the ALLAML dataset, with smaller feature subsets, the classification effect of C4.5 classifier is better. For example, in the warpAP10P dataset, with more feature subsets, C4.5 classifies better.

### 4.4 Comparison the optimal values of algorithms

This section reports the ACC and MCC of the C4.5 classifier on the optimal features selected by all algorithms on 7 datasets for comparison. Tables 3 and 4 report the ACC and MCC of the C4.5 classifier, respectively, obtained with the optimal features selected by all algorithms. Tables 3 and 4 report the ACC and MCC of the Adaboost classifier obtained with the optimal features selected by all algorithms.

The comparison of ACC and MCC of the C4.5 classifier on the optimal features selected by all algorithms in Tables 3 and 4 show that the classification performance of the C4.5 classifier on the features selected by the CGUFS algorithm is significantly better than features selected by existing algorithms on most datasets such as warpPIE10P. On the dataset GLIOMA, the classification performance of the C4.5 classifier on the features selected by IUFS and FSASL algorithms is slightly better than that of features selected by the CGUFS algorithm. On the dataset ALLAML, the classification performance of the C4.5 classifier on the features selected by UDFS algorithm is equivalent to that of the CGUFS algorithm. In addition, the classification performance of the C4.5 classifiers on the features selected by Laplacian, UDFS and SCUFS algorithms is worse than that of the baseline. It indicates that the original dataset contains a lot of redundant and noisy features. Overall, the classification performance of the C4.5 classifier on the features selected by the CGUFS algorithm is superior to all existing algorithms and the basline.

The comparison of ACC and MCC of the Adaboost classifier on the optimal features selected by all algorithms in Tables 5 and 6 shows that, on datasets such as ALLAML, the classification performance of the Adaboost classifier on the features selected by the CGUFS algorithm is significantly better than that of features selected by all existing algorithms. Similarly, the classification performance of the Adaboost classifier on the features selected by Laplacian and SCUFS algorithms is worse than that of the baseline. This indicates that the features selected by Laplacian and UDFS algorithms lead to poor classification ability no matter which classifier is used. However, on the dataset GLIOMA, the Adaboost classifier on the features selected by the IUFS and FSASL algorithms has slightly better classification performance than that of the CGUFS algorithm. In conclusion, no matter which classifier is used, the features selected by the CGUFS algorithm leads to the strongest classification ability.

Therefore, based on the experimental results in Tables 3–6, it can be concluded that no matter which classifier is used to test the selected features, the classification performance on the features selected by the CGUFS algorithm is better than that of the existing algorithms. The IUFS algorithm is the best among all existing algorithms to select representative features. In addition, The FSASL and IUFS algorithms based on clustering guided are obviously superior to Laplaican algorithm.

### 4.5 Comparison the feature scale of algorithms

This section will compare the FS and ACC/FS when the features selected by all algorithms on the 7 datasets are optimal. Tables 7 and 8 reports the FS and ACC/FS of the C4.5 classifier, respectively. Similarly, we also utilize the Adaboost classifier to evaluate the selected features. Tables 9 and 10 reports the FS and ACC/FS of the Adaboost classifier with optimal features selected by all algorithms.

The comparison of FS and ACC/FS of the C4.5 classifier on the optimal features selected by all algorithms in Tables 6 and 7 show that, on some datasets such as Madelon, the FS on features selected by the CGUFS algorithm is significantly smaller than that of all existing algorithms. On some datasets such as Colon, the FS on features selected by the CGUFS algorithm is significantly larger than that of all the existing algorithms. On the dataset ALLAML, the FS on features selected by the CGUFS algorithm is moderate. Overall, optimal features selected by the UDFS algorithm leads to the smallest FS among the existing algorithms, while the FS on features selected by the CGUFS algorithm is in the centered. However, the ACC/FS of the CGUFS algorithm is higher than the UDFS algorithm.

The comparison of FS and ACC/FS of Adaboost classifier on the optimal features selected by all algorithms in Tables 9 and 10 show that, on some datasets such as Madelon, the FS on optimal features selected by the CGUFS algorithm is significantly smaller than that of all existing algorithms. On some datasets such as Colon, the FS on optimal features selected by the DGUFS algorithm has smaller FS, but the ACC and MCC of Adaboost classifier on the selected features are 73.24% and 58.37%, respectively, which are significantly lower than 81:76% and 73:97% of the CGUFS algorithm. In addition, the features selected by the SCUFS algorithm leads to the smallest FS among all existing algorithms, but the ACC and MCC indexes of the Adaboost classifier on the selected features are 71.43% and 60.11% respectively, which are also the worst.

According to the analysis of the scale on the optimal features selected by all algorithm shown in Tables 7–10, no matter which classifier is used to test the selected features, the CGUFS algorithms can select a small number of features that help to obtain the best classification performance of a classifier.

### 4.6 Statistical experiment

This section is the statistical experiment, Wilcoxon and Friedman are selected to test on 7 datasets respectively. First, we use Wilcoxon to perform a pairwise test, using the ACC and MCC indexes of C4.5 and Adaboost on the optimal features selected by all algorithms as data values, with significance level *α* = 0.05, and the null assumption that all feature selection algorithms have the same effect. According to Wilcoxon’s test principle, the test results obtained are shown in Table 10 and 11. According to Wilcoxon’s test principle, the test results obtained are shown in Tables 11 and 12.

The comparison of the ACC and MCC indexes of C4.5 and Adaboost on the optimal features selected by all algorithms in Tables 11 and 12 show that, when the data value is the ACC and MCC indexes of C4.5 classifier show that the maximum *R*– value between the CGUFS and the existing feature selection algorithm is 26 (IUFS). Similarly, when the data value is the ACC and MCC indexes of Adaboost classifier show that the maximum *R*– value between the CGUFS and the existing feature selection algorithm is 39 (FSASL). Since there are 7 datasets and 14 index values, the critical value when *α* = 0.05 is 25, that is, the maximum value for rejecting the null hypothesis is 25. Fortunately, there are significant differences between CGUFS and FSASL algorithms when the classifier is C4.5. Similarly, when the classifier is Adaboost, there are significant differences between CGUFS and IUFS algorithms. In summary, Wilcoxon test based on the ACC and MCC indexes of C4.5 and Adaboost on the optimal features selected by all algorithms shows that there are significant differences between the CGUFS and the existing algorithm.

Further, Friedman is used to perform multiple tests, and also use the ACC and MCC indexes of C4.5 and Adaboost on the optimal features selected by all algorithms as data values. When the significance level *α* = 0.05, the significance of the CGUFS algorithm is tested. The null hypothesis is that all feature selection algorithms have the same effect. When N = 7 and k = 8, Friedman statistical result based on the ACC and MCC indexes of C4.5 classifier is: $\chi_{F}^{2}=32.97$, *F_F_* = 6:59. Similarly, Friedman statistical results based on the ACC and MCC indexes of Adaboost classifier is: $\chi_{F}^{2}=28.10$, *F_F_* = 5.22. When *α* = 0.05, *F*(7, 42) = 2.237. It can be seen that *F_F_* > 2.237 for all indexes. Therefore, Friedman test based on the ACC and MCC indexes of C4.5 and Adaboost on the optimal features selected by all algorithms shows that there are significant differences between the CGUFS and the existing algorithm.

## 5 Discussion

Through the mining of gene expression data, it is helpful to discover the pathogenesis, risk factors and their interactions related to the disease, and provide reference for the clinical diagnosis and treatment of the disease. However, due to the high-dimensional feature space and high feature redundancy of gene expression data, traditional data mining algorithms face problems such as high time complexity and poor classification effect when analyzing gene expression data. Therefore, this paper proposes a cluster-guided unsupervised feature selection algorithm to solve such problem.

It is noted that the CGUFS algorithm has three innovations: (1) An adaptive *k*-means is proposed to group samples. Based on the working principle of the elbow method, when the inflection point is not obvious, we select the *k* with the largest reduction in the sum of squares of error as the optimal *k* value. Similarly, when the inflection point is negative, we select *k* before the inflection point is negative as the optimal *k* value. (2) The spectral clustering is used to group the features. Based on the principle of spectral clustering, all features are grouped so that the correlation between the features within the group is extremely high and the correlation between the features between the groups is extremely low. In addition, the time complexity of feature selection is reduced effectively by dividing features into multiple groups. (3) Potentially efficient and potentially redundant strategies are proposed for feature selection. The potentially effective and potentially redundant strategies are used to calculate the features that need to be retained and deleted in each group, and the number of features that need to be retained is determined adaptively.

In feature selection, embedded algorithms are better than filter algorithms. However, on some datasets, the performance of filter algorithms is lower than embedded algorithms. This result shows that traditional filter algorithms are unstable and may depend on the performance of specific datasets. For example, as shown in Tables 3 and 4, the filter algorithm (i.e. Laplacian) has better ACC and MCC in the Aadelon dataset than most embedded algorithms. In addition, we also evaluate the impact of different classification algorithms on the CGUFS algorithm. The results show that the CGUFS algorithm is effective on different classification algorithms and does not depend on the classification algorithm. Unfortunately, the feature scale selected by the CGUFS algorithm is worse than Laplacian and UDFS. This may be due to the fact that the CGUFS algorithm does not completely delete the redundant features in the gene expression data, so the selected feature scale is not optimal. Therefore, in future research, we will pay more attention to the interaction between features in gene expression data.

## 6 Conclusion and future work

This paper proposes a novel clustering-guided unsupervised feature selection algorithm (CGUFS) specifically designed for the inherent high dimensional feature space and high feature correlation in gene expression data. Different from the traditional unsupervised feature selection algorithm, our proposed algorithm first uses a novel adaptive *k*-means algorithm to group all the samples, obtains the optimal *k* value through several iterations, and assigns it to the corresponding samples. Then, the spectral clustering algorithm is used to group all the features, and the improved *k*-means algorithm is used to get the optimal *k* value and assign it to the corresponding features. Finally, the potential effective feature and potential redundant feature strategy are proposed to identify the potential effective features and potential redundant features in each feature group, and the number of features to be retained in each feature group is calculated according to the feature group strategy. Experimental results show that the classifiers on the optimal features selected by the CGUFS algorithm have significantly better classification ability than that of the existing algorithms, and have less FS. In addition, Friedman tests based on the C4.5 and Adaboost classifiers show significant differences between the CGUFS algorithm and the existing algorithm. However, the feature scale selected by the CGUFS algorithm is not optimal. In the future, we will study how to reduce the selected feature scale without reducing the accuracy of the selected feature subset. In addition, we will also study the application of unsupervised feature selection algorithm in other fields.

## Figures and Tables

**Table 1:** the symbols and definitions used in this paper.

Symbol	Definition	Symbol	Definition
*J*	sum of squared errors	*W*	Similarity matrix
*H*	Indicator vector matrix	*G()*	Undirected graph
*A,B*	The subgraph after Ncut	*H*	Indicator matrix
*L*	Laplacian matrix	*D*	Degree matrix
*V*	Eigenvector matrix	*Y*	Degree vector matrix
*SU()*	Symmetric uncertainty value	*F_j_*	The j-th feature group
*IG()*	Information gain value	$F_{j}^{p}$	The *p*-th feature

**Table 2:** The details of the dataset.

Dataset	Feature	Sample	Class	Feature/Sample
*ALLAML*	7129	72	2	99.01
*GLIOMA*	4434	50	4	88.68
*warpPIE10P*	2420	210	10	11.52
*warpAR10P*	2400	130	10	18.46
*Colon*	2000	62	2	32.26
*Yale*	1024	165	15	6.21
*Madelon*	500	2600	2	0.19

**Table 3:** The optimal features selected by all algorithms corresponds to the ACC index of C4.5 classifier.

Dataset	Basline	Laplacian	MCFS	UDFS	FSASL	SCUFS	IUFS	DGUFS	CGUFS
*ALLAML*	79.2	66.7(8)	77.8(6)	**84.7**(1.5)	80.6(4)	81.9(3)	76.4(7)	79.2(5)	**84.7**(1.5)
*GLIOMA*	58.0	54.0(7.5)	58.0(5)	54.0(7.5)	56.0(6)	64.0(3)	**80.0**(1)	62.0(4)	66.0(2)
*warpAR10P*	70.0	46.2(7)	53.9(5.5)	53.9(5.5)	66.2(2)	43.1(8)	60.0(3)	54.6(4)	**67.7**(1)
*warpPIE10P*	79.1	76.7(7)	81.0(3)	80.5(4)	**84.8**(1)	47.6(8)	84.3(2)	79.5(5)	79.1(6)
*Colon*	82.3	77.4(6)	85.5(2.5)	79.0(5)	85.5(2.5)	75.8(7)	83.9(4)	67.7(8)	**87.1**(1)
*Yale*	48.5	43.6(8)	50.9(3)	45.5(6.5)	53.3(2)	45.5(6.5)	49.7(4)	47.3(5)	**55.2**(1)
*Madelon*	69.0	**80.8**(2)	73.2(5)	72.5(6)	67.9(7)	**80.8**(2)	74.9(4)	57.7(8)	**80.8**(2)
*Average*	69.44	63.63(6.50)	68.61(4.29)	67.16(5.14)	70.61(3.50)	62.67(5.36)	72.74(3.29)	64.00(5.57)	**74.37**(2.07)

**Table 4:** The optimal features selected by all algorithms corresponds to the MCC index of C4.5 classifier.

Dataset	Basline	Laplacian	MCFS	UDFS	FSASL	SCUFS	IUFS	DGUFS	CGUFS
*ALLAML*	53.6	26.5(8)	50.1(6)	**66.6**(1.5)	59.1(3)	59.0(4)	49.5(7)	53.6(5)	**66.6**(1.5)
*GLIOMA*	42.6	36.5(8)	44.3(5)	38.6(7)	39.8(6)	50.0(3)	**72.8**(1)	47.3(4)	53.7(2)
*warpAR10P*	66.9	40.6(7)	53.2(4)	48.9(6)	62.7(2)	36.9(8)	55.1(3)	50.0(5)	**64.3**(1)
*warpPIE10P*	77.0	74.3(7)	79.1(3)	78.6(4)	**83.2**(1)	42.3(8)	82.9(2)	77.6(5)	76.9(6)
*Colon*	60.4	49.8(6)	67.7(3)	55.9(5)	68.0(2)	46.6(7)	64.1(4)	32.6(8)	**71.8**(1)
*Yale*	45.8	39.7(8)	48.6(3)	41.3(7)	51.2(2)	42.3(6)	46.7(4)	44.6(5)	**52.0**(1)
*Madelon*	38.1	**63.6**(1.5)	46.4(5)	44.9(6)	35.8(7)	**63.6**(1.5)	49.7(4)	15.4(8)	61.6(3)
*Average*	54.91	47.29(6.50)	55.63(4.14)	53.54(5.21)	57.11(3.29)	48.67(5.36)	60.11(3.57)	45.87(5.71)	**63.84**(2.21)

**Table 5:** The optimal features selected by all algorithms corresponds to the ACC index of Adaboost classifier.

Dataset	Basline	Laplacian	MCFS	UDFS	FSASL	SCUFS	IUFS	DGUFS	CGUFS
*ALLAML*	87.5	66.7(8)	86.1(2.5)	83.3(6)	84.7(4.5)	86.1(2.5)	79.2(7)	84.7(4.5)	**90.3**(1)
*GLIOMA*	54.0	58.0(7.5)	62.0(5.5)	58.0(7.5)	62.0(5.5)	**74.0**(2)	**74.0**(2)	**74.0**(2)	68.0(4)
*warpAR10P*	92.9	71.4(7)	**95.2**(1.5)	**95.2**(1.5)	76.9(6)	68.6(8)	94.3(4)	92.9(5)	94.8(3)
*warpPIE10P*	83.8	55.4(7)	69.2(4.5)	75.4(3)	76.9(2)	46.2(8)	69.2(4.5)	68.5(6)	**79.2**(1)
*Colon*	77.4	82.3(5)	82.3(5)	82.3(5)	83.9(2)	82.3(5)	82.3(5)	77.4(8)	**88.7**(1)
*Yale*	66.7	55.2(8)	62.4(4.5)	62.4(4.5)	**69.1**(1)	57.0(7)	64.2(3)	57.6(6)	65.5(2)
*Madelon*	67.0	**85.8**(2)	72.1(5)	72.0(6)	66.5(7)	**85.8**(2)	72.8(4)	57.6(8)	**85.8**(2)
*Average*	75.61	67.83(6.36)	75.61(4.07)	75.51(4.79)	74.29(4.00)	71.43(4.93)	76.57(3.86)	73.24(6.00)	**81.76**(2.00)

**Table 6:** The optimal features selected by all algorithms corresponds to the MCC index of Adaboost classifier.

Dataset	Basline	Laplacian	MCFS	UDFS	FSASL	SCUFS	IUFS	DGUFS	CGUFS
*ALLAML*	72.2	23.8(8)	68.9(2)	62.7(6)	66.0(5)	68.7(3)	52.5(7)	68.5(4)	**78.4**(1)
*GLIOMA*	37.0	41.8(8)	49.1(6)	42.5(7)	64.0(2)	60.3(4)	**70.5**(1)	61.7(3)	57.9(5)
*warpAR10P*	82.1	51.3(7)	65.7(5)	72.7(3)	74.4(2)	40.5(8)	66.6(4)	64.9(6)	**77.3**(1)
*warpPIE10P*	92.1	68.2(7)	94.8(2.5)	94.8(2.5)	**95.7**(1)	65.3(8)	93.7(5)	92.2(6)	94.2(4)
*Colon*	49.1	60.9(4)	60.4(6)	60.9(4)	64.0(2)	60.9(4)	60.2(7)	50.7(8)	**75.3**(1)
*Yale*	64.4	52.7(8)	60.7(4)	60.0(5)	**67.3**(1)	53.5(7)	62.6(3)	55.4(6)	63.1(2)
*Madelon*	34.0	**71.6**(2)	44.2(5)	44.4(6)	33.0(7)	**71.6**(2)	45.6(4)	15.2(8)	**71.6**(2)
*Average*	61.56	52.90(6.29)	63.40(4.36)	62.57(4.79)	66.34(2.86)	60.11(5.14)	64.53(4.43)	58.37(5.86)	**73.97**(2.29)

**Table 7:** The FS of C4.5 classifier on the optimal features selected by all algorithms.

Dataset	Basline	Laplacian	MCFS	UDFS	FSASL	SCUFS	IUFS	DGUFS	CGUFS
*ALLAML*	7129	**10**	90	40	30	80	**10**	100	60
*GLIOMA*	4434	**10**	130	20	40	30	80	80	**10**
*warpAR10P*	2420	80	80	60	100	130	**30**	110	130
*warpPIE10P*	2400	140	150	100	110	150	150	**70**	100
*Colon*	2000	90	70	130	70	10	50	**20**	150
*Yale*	1024	140	130	90	70	130	150	130	**60**
*Madelon*	500	20	80	60	150	20	90	110	**10**
*Average*	2843.86	70.00	104.29	**71.43**	81.43	78.57	80.00	88.57	74.29

**Table 8:** The ACC/FS of C4.5 classifier on the optimal features selected by all algorithms.

Dataset	Basline	Laplacian	MCFS	UDFS	FSASL	SCUFS	IUFS	DGUFS	CGUFS
*ALLAML*	0.01	6.67	0.86	2.12	2.69	1.02	**7.64**	0.07	1.41
*GLIOMA*	0.01	5.40	0.45	2.70	1.40	2.13	1.33	0.78	**6.60**
*warpAR10P*	0.04	0.96	1.01	1.34	0.85	0.37	**2.81**	0.70	0.61
*warpPIE10P*	0.03	0.33	0.36	0.54	0.60	0.29	0.40	**0.78**	0.68
*Colon*	0.04	0.86	1.22	0.61	1.22	7.58	1.68	**3.39**	0.58
*Yale*	0.05	0.31	0.39	0.51	0.76	0.35	0.33	0.36	**0.93**
*Madelon*	0.14	1.04	0.92	1.21	0.45	4.04	0.83	0.52	**8.08**
*Average*	0.02	0.91	0.66	0.94	0.87	0.80	0.91	0.72	**1.00**

**Table 9:** The FS of Adaboost classifier on the optimal features selected by all algorithms.

Dataset	Basline	Laplacian	MCFS	UDFS	FSASL	SCUFS	IUFS	DGUFS	CGUFS
*ALLAML*	7129	140	130	140	40	90	10	150	**50**
*GLIOMA*	4434	40	110	130	**20**	70	80	70	80
*warpAR10P*	2420	150	120	140	110	140	140	**100**	150
*warpPIE10P*	2400	**110**	150	150	130	140	140	150	**110**
*Colon*	2000	90	70	140	130	**50**	110	70	150
*Yale*	1024	140	140	140	**130**	100	**130**	140	150
*Madelon*	500	**20**	130	60	150	**20**	90	110	**20**
*Average*	2843.86	98.57	121.43	128.57	101.43	**87.14**	100.00	112.86	101.43

**Table 10:** The ACC/FS of Adaboost classifier on the optimal features selected by all algorithms.

Dataset	Basline	Laplacian	MCFS	UDFS	FSASL	SCUFS	IUFS	DGUFS	CGUFS
*ALLAML*	0.01	0.48	0.66	0.60	2.12	0.96	7.92	0.56	**1.81**
*GLIOMA*	0.01	1.45	0.56	0.45	**3.10**	1.06	0.93	1.06	0.85
*warpAR10P*	0.04	0.48	0.79	0.68	0.87	0.49	0.67	**0.93**	0.63
*warpPIE10P*	0.03	0.50	0.46	0.50	0.59	0.33	0.49	0.46	**0.72**
*Colon*	0.04	0.91	**1.18**	0.59	0.65	1.65	0.75	1.11	0.59
*Yale*	0.07	0.39	0.45	0.45	0.53	**0.57**	0.49	0.41	0.44
*Madelon*	0.13	**4.29**	0.55	1.20	0.44	**4.29**	081	0.52	**4.29**
*Average*	0.03	0.69	0.62	0.59	0.73	0.82	0.77	0.65	**0.81**

**Table 11:** Wilcoxon test based on the ACC and MCC indexes of C4.5 on the optimal features selected by all algorithms.

Dataset	Laplacian	MCFS	UDFS	FSASL	SCUFS	IUFS	DGUFS
*R*+	17.5	15	19.5	15	17.5	26	15
*R*−	87.5	90	85.5	90	87.5	79	90
*Hypothesis*	Reject	Reject	Reject	Reject	Reject	No Reject	Reject
*Selected*	CGUFS	CGUFS	CGUFS	CGUFS	CGUFS	IUFS	CGUFS

**Table 12:** Wilcoxon test based on the ACC and MCC indexes of Adaboost on the optimal features selected by all algorithms.

Dataset	Laplacian	MCFS	UDFS	FSASL	SCUFS	IUFS	DGUFS
*R*+	10.5	14	14	39	21.5	11	11
*R*−	94.5	91	91	66	83.5	94	94
*Hypothesis*	Reject	Reject	Reject	No Reject	Reject	Reject	Reject
*Selected*	CGUFS	CGUFS	CGUFS	FSASL	CGUFS	CGUFS	CGUFS

## Data Availability

The datasets used can be accessed freely from http://featureselection.asu.edu/datasets.php. The code and other materials during the current study can be available from the co-corresponding authors on reasonable request.
